# Photodriven Methane Conversion on Transition Metal Oxide Catalyst: Recent Progress and Prospects

**DOI:** 10.1002/advs.202305471

**Published:** 2023-10-26

**Authors:** Pu Wang, Run Shi, Jiaqi Zhao, Tierui Zhang

**Affiliations:** ^1^ Key Laboratory of Photochemical Conversion and Optoelectronic Materials Technical Institute of Physics and Chemistry Chinese Academy of Sciences Beijing 100190 China; ^2^ Center of Materials Science and Optoelectronics Engineering University of Chinese Academy of Sciences Beijing 100049 China

**Keywords:** C–H activation, photocatalysis, semiconductor, transition metal oxide

## Abstract

Methane as the main component in natural gas is a promising chemical raw material for synthesizing value‐added chemicals, but its harsh chemical conversion process often causes severe energy and environment concerns. Photocatalysis provides an attractive path to active and convert methane into various products under mild conditions with clean and sustainable solar energy, although many challenges remain at present. In this review, recent advances in photocatalytic methane conversion are systematically summarized. As the basis of methane conversion, the activation of methane is first elucidated from the structural basis and activation path of methane molecules. The study is committed to categorizing and elucidating the research progress and the laws of the intricate methane conversion reactions according to the target products, including photocatalytic methane partial oxidation, reforming, coupling, combustion, and functionalization. Advanced photocatalytic reactor designs are also designed to enrich the options and reliability of photocatalytic methane conversion performance evaluation. The challenges and prospects of photocatalytic methane conversion are also discussed, which in turn offers guidelines for methane‐conversion‐related photocatalyst exploration, reaction mechanism investigation, and advanced photoreactor design.

## Graphical abstract

Photocatalytic methane conversion is a promising route for converting abundant and affordable methane into value‐added products driven by clean sustainable light energy under mild conditions. In this paper, the research progress of photocatalytic methane conversion, including methane activation mechanism, methane conversion reaction, and photocatalytic reactor design is reviewed, and the challenges and development prospects for photocatalytic methane conversion are discussed.

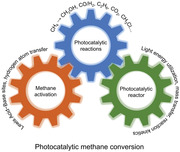

## 1 Introduction

**Figure 1:**
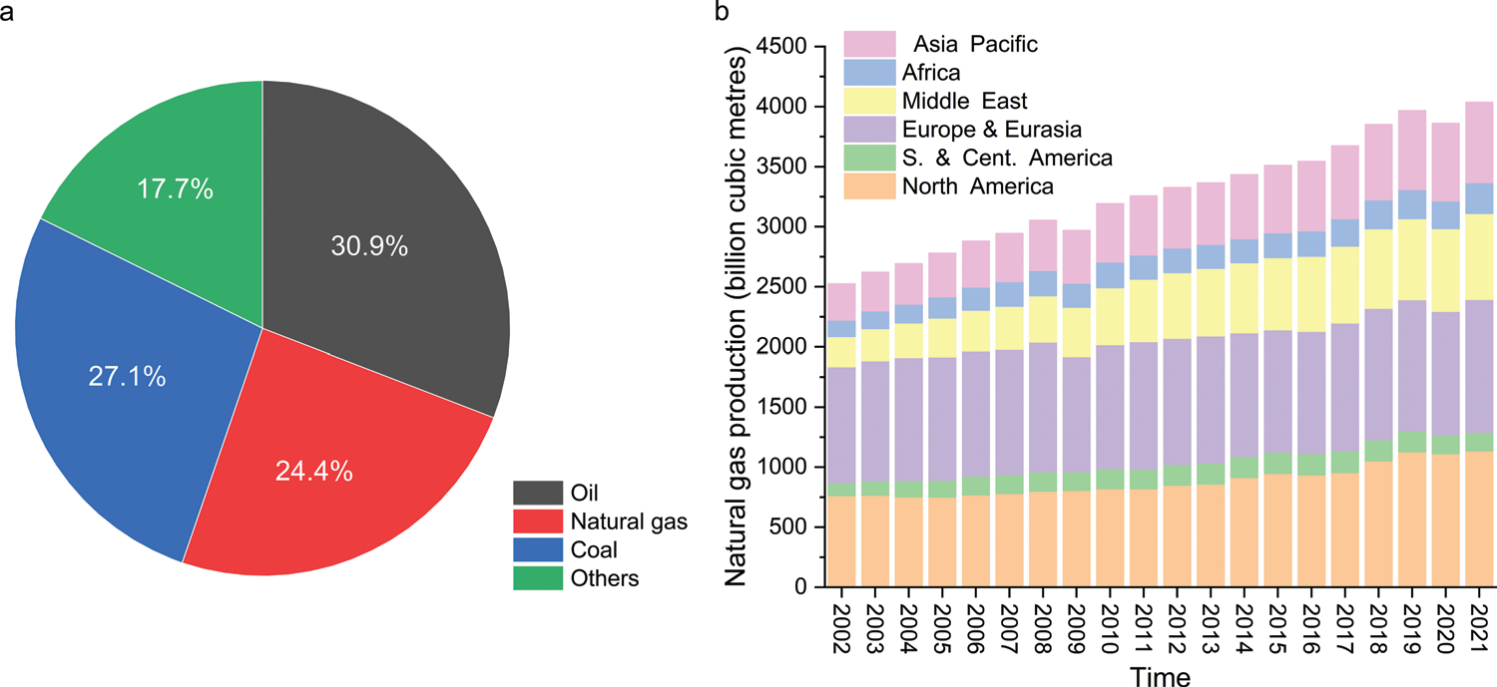
a) Distribution of global energy production in 2021, showcasing the shares of natural gas, coal, oil and other energy sources such as wind, nuclear, and biomass. b) Natural gas production trends in various regions worldwide over the last 20 years. The data is reprinted from the BP Statistical Review of World Energy 2022.^[^ ^1^ ^]^

Natural gas, which includes shale gas, methane hydrate and coal‐bed methane, plays an irreplaceable role as energy source in human society. In 2021, it accounted for 24.4% of the global total energy production (**Figure** **1**a). Over the past 20 years, global natural gas production has increased from 2523.9 billion cubic meters to 4036.9 billion cubic meters, representing a remarkable growth of approximately 60% (Figure 1b).^[^ ^1^ ^]^ Compared with oil and coal, natural gas combustion releases significantly fewer sulfur oxide and nitrogen oxide pollutants while offering a much higher calorific value, making it the cleanest fossil energy source.^[^ ^2^, ^3^ ^]^

From a global perspective, the development of C_1_ chemistry using natural gas as raw material has emerged as a prominent research hotspot. It is anticipated that the chemical industry is poised to enter an era of rapid development with natural gas playing a pivotal role in the near future.^[^ ^4^ ^]^ With the proven reserves of natural gas continuously increasing, methane, as the predominant main component, has garnered significant attention as a potential chemical feedstock instead of solely being used as fuel. This shift offers a glimpse of reducing the current chemical industry's heavy reliance on increasingly depleted crude oil.^[^ ^5^, ^6^, ^7^ ^]^

Catalytic methane conversion is an important means to achieve the conversion of methane to valuable chemicals.^[^ ^8^ ^]^ However, owing to the high bond energy and low polarizability of the C–H bond, methane conversion often requires harsh reaction conditions to activate methane activation, leading to energy consumption, catalyst deactivation, and side reactions. For example, Ni–Mo nanocatalysts on single‐crystalline MgO is used for thermocatalytic methane dry reforming but accompanied by irrepressible agglomeration of NiMo alloys at 800 °C.^[^ ^9^ ^]^ In addition, currently only methane reforming to syngas has achieved large‐scale industrial applications through steam methane reforming, and the resultant syngas is subsequently used in large quantities for the synthesis of methanol, light olefins, and liquid hydrocarbons.^[^ ^10^ ^]^ Unfortunately, such conversion route increases process complexity and energy consumption due to dependence on high temperatures and pressures. Direct conversion of methane under mild conditions is an ideal target but is mostly limited by thermodynamics.

The emerging photocatalytic methane conversion, characterized by high‐energy excited states under light irradiation, breaks the thermodynamic limitation with clean and sustainable solar energy as the driving force under mild conditions.^[^ ^11^, ^12^, ^13^ ^]^ In the past few years, photocatalytic methane conversion under mild conditions has gained increasing attention, which has pushed forward the development of photocatalytic methane conversion. Zeng et al. conducted a systematic review of the band structure and charge carrier transfer in semiconductor nanomaterials for photocatalytic methane oxidation, and categorized the progress based on the involved oxidizing agents.^[^ ^14^ ^]^ Additionally, there have been summaries of historical development of photocatalysts and cocatalysts used in photocatalytic methane conversion.^[^ ^15^, ^16^ ^]^ Considering the significant advancements in transition metal oxide‐based nanomaterials for methane photoactivation and selective conversion, it becomes essential to provide a comprehensive overview the state‐of‐the‐art photocatalysts and corresponding photoreactors that have been recently developed for various photocatalytic methane conversion reactions.

In this review, we aim to provide a comprehensive overview of recent developments in photocatalytic methane conversion. Firstly, we place significant emphasis on understanding the basis of methane activation. Subsequently, we categorize the major methane conversion reactions, encompassing photocatalytic partial oxidation of methane (methane‐to‐methanol), methane reforming (methane‐to‐syngas), methane coupling (methane‐to‐C_2+_ hydrocarbon), methane combustion (methane‐to‐CO_2_), and methane functionalization (construction of C–Cl, C–B, and C–S bonds). Additionally, we provide a summary of photoreactors to provide new options for photocatalytic performance testing and future applications. Finally, we address challenges and prospects to inspire further advancements in the field of photocatalytic methane conversion.

## 2 Fundamentals of Photocatalytic Methane Conversion

**Figure 2:**
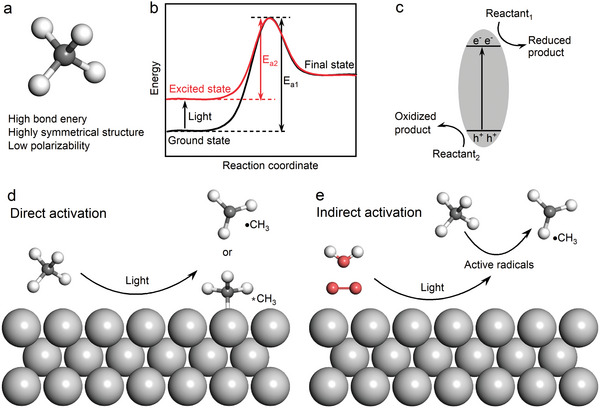
a) Molecular structure of methane. b) Energy changes in ground state and excited state reactions. c) Basic principles of photocatalytic reactions. d) Direct activation of methane on the photocatalyst surface. e) Indirect activation of methane by light‐induced active radicals.

Methane exhibits high chemical inertness due to its symmetrical tetrahedral structure, characterized by four equal C–H bonds, which imparts it with high bond energy, low electron and proton affinity, and low polarizability (**Figure** **2**a).^[^ ^17^, ^18^ ^]^ The relatively large gap between the highest occupied molecular orbital (HOMO) and the high lowest unoccupied molecular orbital (LUMO) in methane creates challenges for the molecules to acquire electrons for reduction or to release electrons for oxidation.^[^ ^8^, ^19^ ^]^ This inherent inertness of methane's structure leads to the need to overcome an extremely high activation energy barrier (*E*_a_) during its thermocatalytic conversion, necessitating the use of high temperatures (700—1000 °C) for efficient methane conversion. Given such harsh reaction conditions, the thermocatalytic methane conversion is energy intensive with high carbon emissions despite decades of considerable effort being invested in efficient methane conversion systems centered on the development of advanced catalysts. The only well‐established methane conversion route is indirect, where methane is first steam reformed to syngas, which is then used to synthesize light olefins, methanol and liquid hydrocarbons. The effective conversion of methane into a range of carbon‐containing chemicals, such as alcohols, aromatics, long‐chain alkanes and olefins, has long been considered the holy grail in fields of catalysis and chemistry.^[^ ^20^ ^]^

Photocatalysis, which can directly converts solar energy into chemical energy, stands as one of the most ideal approaches to drive methane conversion.^[^ ^21^, ^22^ ^]^ When exposed to ultraviolet light or visible light photons, the photocatalyst becomes excited to a high‐energy excited state, altering the reaction path and reducing the activation energy of the reaction (Figure 2b). Photocatalysis enables the disruption of thermodynamic equilibrium, allowing for even thermodynamic uphill reactions to occur under mild conditions.^[^ ^23^, ^24^ ^]^ In this process, photogenerated electrons are excited to the conduction band to facilitate the reduce half‐reaction, while the oxidation half‐reaction is carried out by the photogenerated holes left in the valence band (Figure 2c).

Following the electron–hole separation and transfer process, heterogeneous photocatalysts primarily involve processes occurring at the catalyst–medium interface, encompassing the adsorption and activation of reactants, formation of intermediates, and desorption and desorption of products.^[^ ^25^, ^26^, ^27^ ^]^ The complicated reaction processes at the catalytic interface pose challenges in finely regulating the overall efficiency of the catalytic reaction.^[^ ^28^, ^29^ ^]^ Methane activation is frequently regarded as the rate‐determining step in methane conversion reactions.^[^ ^30^, ^31^ ^]^ Photocatalytic methane activation based on heterogeneous catalysts can be classified into two types: 1) direct activation occurs when methane is directly adsorbed and activated on the photocatalyst surface under illumination (Figure 2d); 2) indirect activation take place when methane is activated with the assistance of light‐indued reactive radicals originating from additional reactants, such as water and oxygen molecules (Figure 2e). Despite the differences between the two methane activation pathways, light‐associated reactive oxygen species play key roles in both the two pathways including the production of photogenerated surface active sites (e.g., O^−^ on oxides) and active radicals (e.g., •OH and •O_2_^−^).

**Figure 3:**
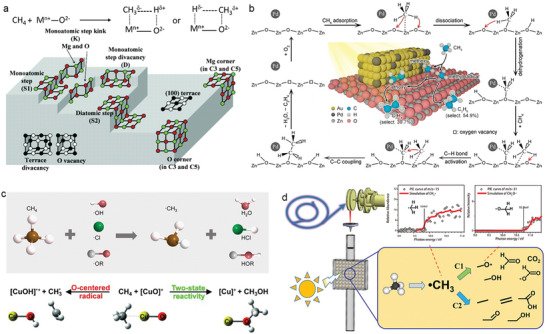
a) Methane adsorption and C–H bond polarization on the basic metal oxide surface and a schematic representation of different sites on the surface of MgO. Reproduced with permission.^[^ ^38^, ^39^ ^]^ Copyright 2017 and 2006, American Chemical Society. b) Schematic illustration of the photocatalytic conversion of CH_4_ to C_2_H_4_ through surface alkoxy intermediates in the case of ZnO‐AuPd hybrid catalysts. Reproduced with permission.^[^ ^40^ ^]^ Copyright 2021, American Chemical Society. c) Schematic illustration of methane activation by hydrogen atom transfer. Top: Reproduced with permission.^[^ ^41^ ^]^ Copyright 2023, Chinese Chemical Society. Bottom: Reproduced with permission.^[^ ^44^ ^]^ Copyright 2011, Wiley‐VCH. d) Detection of reactive intermediates detection during photocatalytic methane oxidation reactions through in situ synchrotron radiation photoionization mass spectrometry. Reproduced with permission.^[^ ^45^ ^]^ Copyright 2023, Wiley‐VCH.

For direct activation routes, acid/base sites and crystal defects engineering are commonly employed modification strategies to optimize the photocatalytic methane activation process.^[^ ^32^, ^33^, ^34^ ^]^ Basic sites on metal oxides are believed to play a crucial role in methane activation owing to their relatively strong electronic interaction with weakly acidic methane.^[^ ^35^, ^36^, ^37^ ^]^ Methane adsorbed on the metal oxide results in the formation of a negatively charge fragment coordinated to the metal cation and a positively charged one coordinated to the basic lattice oxygen of the metal oxide, leading to C–H bond polarization on the metal oxide surface (**Figure** **3**a). Depending on the level of basicity the metal oxide, the two parts will demonstrate either a Lewis acid (positive) or a Lewis base (negative).^[^ ^38^ ^]^ Besides, defects in nanocatalysts result in the disruption of translational symmetry in crystal cells, including 3D volumetric defects (such as pores), 2D planar defects (such as grain boundaries), 1D linear defects (dislocations) and 0D point defects (such as vacancies). These defects directly influence the coordination structure and corresponding catalytic properties of metal oxides.^[^ ^39^ ^]^ For example, the Pd‐modified ZnO–Au composite possesses a unique interface defect structure that promotes the dissociation of CH_4_ molecules to methoxyl and methyl groups, facilitating the photocatalytic methane‐ethylene conversion (Figure 3b).^[^ ^40^ ^]^

For indirect routes, hydrogen atom transfer is an effective strategy for activating methane C–H bonds. It is an elementary reaction that involves the transfer of hydrogen atoms through the co‐transfer of protons and electrons in radical reactions (Figure 3c).^[^ ^41^, ^42^ ^]^ Photocatalytic hydrogen atom transfer utilizes free radical intermediates produced during photocatalysis to activate C–H bonds.^[^ ^43^ ^]^ Bare (CuO)^+^ cation in the gas phase can interact with oxygen‐centered free radicals and facilitate the process of hydrogen atoms transfer and methane activate.^[^ ^44^ ^]^ An in situ photocatalytic mass spectrometry technique based on synchrotron radiation has been reported to capture active gas‐phase intermediates in photocatalytic methane oxidation reactions.^[^ ^45^ ^]^ In addition to several stable species like CO_2_, H_2_O, C_2_H_6_, and CH_3_OH, active methyl radicals (•CH_3_) and methoxy radicals (CH_3_O•) have also been successfully detected (Figure 3d). Moreover, mass spectrometry combined with quantum chemical calculations revealed that the (AuO)^+^ activates methane to methanol through selective oxygen atoms transfer from (AuO)^+^, rather than extracting hydrogen atoms transfer of hydrogen atoms from methane, although the latter one was observed in the congeners (CuO)^+^ and (AgO)^+^.^[^ ^46^ ^]^

**Figure 4:**
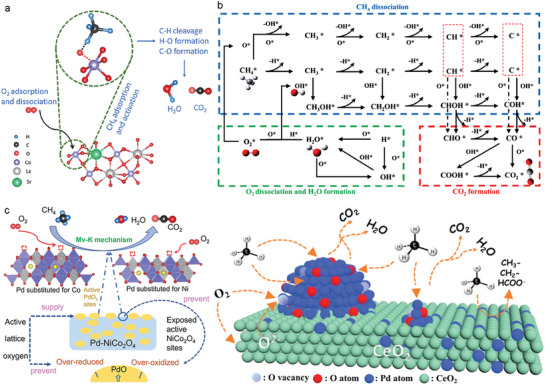
a) Schematic diagrams of the proposed suprafacial Rideal–Eley mechanism for lanthanum, cobalt‐based perovskite oxide catalysts. Reproduced with permission.^[^ ^47^ ^]^ Copyright 2020, Elsevier. b) Reaction pathways through the Langmuir–Hinshelwood mechanism in the catalytic combustion of methane. Reproduced with permission.^[^ ^48^ ^]^ Copyright 2021, Elsevier. c) Methane oxidation reaction through the Mars–van Krevelen mechanism for Pd incorporated Pd‐NiCo_2_O_4_ and Pd/CeO_2_. Left: Reproduced with permission.^[^ ^50^ ^]^ Copyright 2021, Elsevier. Right: Reproduced with permission.^[^ ^51^ ^]^ Copyright 2021, American Chemical Society.

Oxidative dehydrogenation of methane is another widely investigated indirect route for methane activation and conversion under mild conditions. Depending on the forms of oxygen active species involved, the reaction mainly follows three mechanisms: the Rideal–Eley (R–E) mechanism and Langmuir‐Hinshelwood (L‐H) mechanism, both dominated by surface‐adsorbed oxygen, and the Mars–van Krevelen (Mv–K) mechanism, dominated by lattice oxygen. Here is a brief introduction to the reaction mechanisms:

1) R–E mechanism: CH_4_ molecules initially bind to lattice oxygen in the transition metal oxide, causing the C–H bonds in CH_4_ molecules to break and form free methyl radicals, which are subsequently oxidized.^[^ ^47^ ^]^ Simultaneously, the binding process leads to the consumption of crystal lattice oxygen, resulting in the formation of weakly electric oxygen vacancies. These vacancies absorb molecular oxygen to replenish the lost crystal lattice oxygen to complete the catalytic cycle (**Figure** **4**a).
2) L–H mechanism: The chemosorption of molecular oxygen on the catalyst surface is significantly easier than that of methane, leading to the preferential adsorption of molecular oxygen on the catalyst surface when exposed to oxygen‐containing atmosphere.^[^ ^48^ ^]^ Those adsorbed oxygen species exhibits higher reactivity with CH_4_ molecules compared to molecular oxygen to combine with, causing the elongation of C–H bonds in methane. The elongation subsequently disrupts the regular tetrahedron structure of CH_4_ molecules, generating active methyl radicals and promoting the oxidative dehydrogenation of CH_4_ (Figure 4b). The L–H mechanism is mainly used in noble‐metal‐supported metal oxide catalysts.^[^ ^49^ ^]^
3) The Mv–K mechanism involves both surface oxygen reactions and lattice oxygen migration.^[^ ^50^ ^]^ First, gaseous CH_4_ molecules are adsorbed on the catalyst's active site. The adsorbed CH_4_ molecule is then react with surface lattice oxygen to form CH_3_^+^ ions, which are subsequently oxidized to adsorbed products such as CO_2_, and H_2_O molecules, leading to the creation of oxygen vacancies. This step can be described as catalyst reduction. Finally, the adsorbed products are desorbed, while internal lattice oxygen migrates to the surface, refilling the oxygen vacancies with the surface‐adsorbed oxygen, described as catalyst reoxidation (Figure 4c).

## 3 Photocatalytic Methane Conversion Reactions

### 3.1 Partial Oxidation of Methane

**Figure 5:**
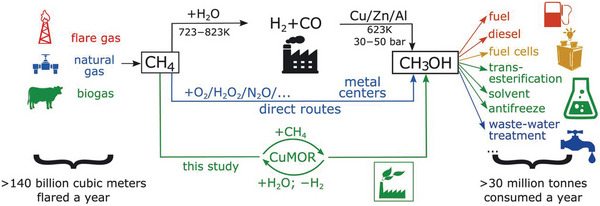
Current and proposed chemical processes for converting methane to methanol. Although the indirect route via syngas requires harsh conditions and involves two separate steps, it is used to produce methanol with nearly 100% selectivity. Direct routes using O_2_, H_2_O_2_, N_2_O and other oxidants show promise with high selectivity and/or efficiency toward methanol. Reproduced with permission.^[^ ^62^ ^]^ Copyright 2017, American Association for the Advancement of Science.

Depending on the reaction products, methane conversion reactions are classified into partial oxidation of methane, methane reforming, methane coupling, and methane combustion.^[^ ^52^ ^]^ Among them, partial oxidation of methane is a promising pathway for producing valuable oxygenates, such as methanol, formaldehyde, and formic acid.^[^ ^53^, ^54^, ^55^ ^]^ Methanol is considered to be the ideal methane conversion product due to its characteristics as an easy‐to‐transport liquid that can be utilized as both fuel and a fundamental chemical feedstock.^[^ ^56^, ^57^, ^58^ ^]^ Conventional methane‐to‐methanol conversion follows an energy‐intensive multistep route, wherein methane is steam reformed at high temperatures to produce syngas (CO and H_2_). The syngas is then utilized to synthesize methanol under high pressure.^[^ ^59^, ^60^, ^61^ ^]^ The one‐step conversion of methane into methanol is an alternative pathway with lower energy consumption.^[^ ^62^, ^63^ ^]^ However, methanol is prone to overoxidation, leading to the production of other by‐products. As a result, it is crucial to enhance methanol selectivity for methane partial oxidation reactions.^[^ ^64^ ^]^ Sushkevich et al. reported a step‐by‐step path using zeolite with copper site as a catalyst based on the partial oxidation of water, enabling anaerobic oxidation of methane to methanol with 97% selectivity (**Figure** **5**). However, the catalyst requires prior activation in 673 K helium and subsequent continuous exposure to 7 bar methane and water at 473 K.^[^ ^62^ ^]^ Xie et al. designed a 2D in‐plane Z‐Scheme heterostructure composed of ZnO and Fe_2_O_3_ porous nanosheets, achieving the one‐step photooxidation of methane with nearly 100% methanol selectivity at room temperature and ambient pressure.^[^ ^65^ ^]^

Methane tends to proceed more thermodynamically easily with the involvement of oxidizing agents such as O_2_, H_2_O_2_, and N_2_O. Among these oxidants, O_2_ is the most prevalent choice for photocatalytic partial oxidation of methane to methanol due to its easy availability and economic superiority (Equation (1))

(1) $$2{\mathrm{CH}}_{4}+O_{2}\to 2{\mathrm{CH}}_{3}\mathrm{OH},\Delta G_{298\;K}^{0}=\;-223{\;\mathrm{kJ}\;\mathrm{mol}}^{-1}$$

**Figure 6:**
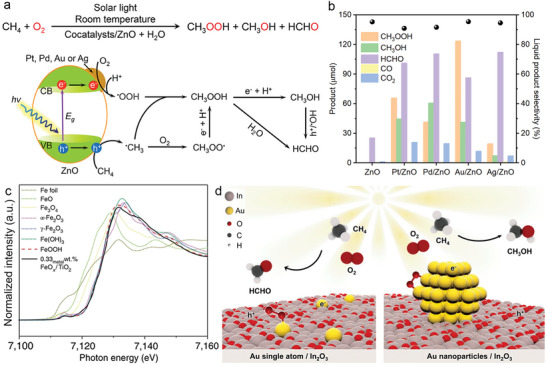
a) Proposed reaction mechanism for photocatalytic CH_4_ oxidation to CH_3_OOH, CH_3_OH, and HCHO over cocatalyst/ZnO. CB, conduction band; VB, valence band; *E*_g_, bandgap energy. b) Photocatalytic activity of cocatalyst/ZnO for the oxidation of CH_4_ using O_2_ as the oxidant. a‐b) Reproduced with permission.^[^ ^67^ ^]^ Copyright 2019, American Chemical Society. c) Fe K‐edge X‐ray absorption near‐edge structure spectra 0.33_metal_wt% FeO*_x_*/TiO_2_ and control samples. Reproduced with permission.^[^ ^69^ ^]^ Copyright 2018, Nature Publishing Group. d) Proposed mechanism of photocatalytic conversion of CH_4_ on Au_1_/In_2_O_3_ and Au_NPs_/In_2_O_3_. Reproduced with permission.^[^ ^70^ ^]^ Copyright 2023, American Chemical Society.

A variety of transition metal oxide‐based photocatalysts have been designed for photocatalytic methane‐to‐methanol conversion at room temperature (**Table** **1**).^[^ ^66^ ^]^ ZnO supported with noble metal cocatalysts (Pt, Pd, Au, or Ag) typically exhibits effective C–H bond activation and controllable molecular oxygen activation under light irradiation, enabling direct oxidization of methane in to methanol and formaldehyde with oxygen and water (**Figure** **6**a).^[^ ^67^ ^]^ Depositing the cocatalysts Pt, Pd, Au, or Ag nanoparticles (NPs) on ZnO with a concentration of 0.1 wt% promoted the production of oxygenated liquid products (CH_3_OOH, CH_3_OH, and HCHO), whereas loading these cocatalysts contributed little to the formation of liquid oxygenates over ZnO (Figure 6b). A unique core‐shell nanostructured photocatalyst, comprising silica‐encapsulated TiO_2_ decorated with AuPd nanoparticles (TiO_2_@SiO_2_‐AuPd), can effectively prevent the overoxidation of methanol and improve the yield of oxygenates even exposed to intensive UV illumination.^[^ ^68^ ^]^Highly dispersed iron oxides (FeO*_x_*) were anchored to a TiO_2_ support to form FeOOH and Fe_2_O_3_ active sites for the photocatalytic methane oxidation to methanol (Figure 6c).^[^ ^69^ ^]^ The efficient electron transport from TiO_2_ to surface iron species serves as the fundamental source of high activity, while Fe_2_O_3_ reduces the energy barrier of H_2_O_2_ reduction on iron species, thus inhibiting overoxidation to CO_2_. By adjusting the band structure and the size of the active site (single atom or nanoparticle) in the Au/In_2_O_3_ photocatalyst, two important free radicals, •OOH and •OH, are formed, which are conducive to the formation of methanol and formaldehyde through different reaction pathways.^[^ ^70^ ^]^ The In_2_O_3_‐supported Au single atom catalyst exhibits a selectivity of 97.62% to formaldehyde with a production yield of 6.09 mmol g^−1^, while the In_2_O_3_‐supported Au nanoparticle catalyst shows a selectivity of 89.42% to methanol with a production yield of 5.95 mmol g^−1^ (Figure 6d). In addition, PdO/Pd‐WO_3_ has been demonstrated to directly convert methane to acetic acid using methane and water as reactants.^[^ ^71^ ^]^ Under the action of photogenerated •OH, methane molecules are activated as methyl radicals and carbonyl intermediates at the Pd and PdO sites, respectively.

**Table 1:** Representative studies on the photocatalytic partial oxidation of methane.

Catalyst	Reactants	Reaction conditions	Performance	Ref.
Au_1_/In_2_O_3_	10 bar O_2_, 20 bar CH_4_, 180 mL H_2_O	10 mg catalyst, Xe lamp (300–1100 nm), 250 mW cm^−2^, 3 h	HCHO: 5.95 mmol g^−1^ CO_2_: 0.15 mmol g^−1^	[70]
Au_NPs_/In_2_O_3_	10 bar O_2_, 20 bar CH_4_, 180 mL H_2_O	10 mg catalyst, Xe lamp (30–1100 nm), 250 mW cm^−2^, 3 h	CH_3_OH :6.09 mmol g^−1^ HCHO: 0.11 mmol g^−1^ CO_2_: 0.58 mmol g^−1^ C_2_H_6_: 0.03 mmol g^−1^	[70]
q‐BiVO_4_	10 bar CH_4_ 10 bar O_2_ 10 mL H_2_O	10 mg catalysts, Hg lamp (300–600 nm), 170 mW cm^−2^, 3 h	CH_3_OH :2.3 mmol g^−1^ HCHO: 1.9 mmol g^−1^ CO_2_: 0.5 mmol g^−1^	[72]
0.1 wt% Au/ZnO	2 MPa CH_4_, 1 MPa O_2_, 100 mL H_2_O	10 mg catalyst, 300 W Xe lamp (300–500 nm), 100 mW cm^−2^, 2 h	CH_3_OOH: 123.4 µmol CH_3_OH :41.2 µmol HCHO: 86.3 µmol CO: 0.4 µmol CO_2_: 11.6 µmol	[67]
Pd–def–In_2_O_3_	19 bar CH_4_, 1 bar O_2_ 50 mL H_2_O	20 mg catalyst, 420 nm LED lamp,3 h	CH_3_OOH: 107.6 µmol CH_3_OH: 37.9 µmol HCHO: 334.3 µmol CO_2_: 1.1 µmol	[73]
Cu–W–TiO_2_	2 MPa CH_4_, 0.2 MPa O_2_, 50 mL H_2_O	20 mg catalyst, 300 W Xe lamp (350–760 nm), 200 mW cm^−2^, 2 h	CH_3_OOH: ≈9.5 mmol g^−1^ CH_3_OH: ≈1 mmol g^−1^ HCHO: ≈5.5 mmol g^−1^ HCOOH: ≈7 mmol g^−1^ CO: ≈0.5 mmol g^−1^ CO_2_: ≈2 µmol g^−1^ h^−1^	[74]
HSiMo/TiO_2_	2 MPa O_2_, 3 MPa CH_4_ 20 mL H_2_O	20 mg catalyst, 300 W Xe lamp,150°C, 2 h	CH_3_OH: 183.2 µmol g^−1^ HCHO: 1344.6 µmol g^−1^ HCOOH: 1359.8 µmol g^−1^ CH_3_CHO: 53.7 µmol g^−1^ CO_2_: 300.9 µmol g^−1^	[75]
Au_0.2_Cu_0.15_–ZnO	1 bar O_2_, 19 bar CH_4_, 100 mL H_2_O	20 mg catalyst, 300 W Xe lamp, 150 mW cm^−2^, 2 h	CH_3_OOH: 4870.4 µmol g^−1^ CH_3_OH: 12 906.4 µmol g^−1^ HCHO: 4673.0 µmol g^−1^ CO_2_: 42.6 µmol g^−1^	[76]
FeO_x_/TiO_2_	70 µmol CH_4_ in Ar, 8 µmol H_2_O_2_ in 10 mL H_2_O	10 mg catalysts, 300 W Xe lamp, 710 nm short pass filter, 3 h	CH_4_ conversion: 14.9% CH_3_OH: 1,056 µmol g_cat_^−1^	[69]
TiO_2_(001)–C_3_N_4_	8%CH_4_, 4%O_2_, 165 µL H_2_O_2_ in 20 mL H_2_O	20 mg catalyst, 300 W Xe lamp, 8 h	CH_3_OOH: 55.8 µmol g^–1^ h^–1^ CH_3_OH: 19.6 µmol g^−1^ h^–1^ CH_3_CH_2_OH: 7.9 µmol g^−1^ h^−1^ HCOOH: 235.0 µmol g^−1^ h^−1^ CH_3_COOH: 72.1 µmol g^−1^ h^−1^ CO*_x_*: 126.2 µmol g^−1^ h^−1^	[77]
2Cu/CeO_2_	1.2 MPa CH_4_ (95% in Ar), 1.2 MPa CO_2_ (99.999%), 50 mL H_2_O	30 mg catalyst, 300 W Xe lamp, 120°C, 3 h	CH_3_OH: 88.9 µmol g^−1^ h^−1^ CH_3_OH selectivity: 96.2%	[78]
ZnO/Fe_2_O_3_	CH_4_, 5 mL H_2_O	300 W Xe lamp, AM 1.5 G	CH_3_OH: 178.3 µmol^−1^ g_cat_^−1^	[65]

**Figure 7:**
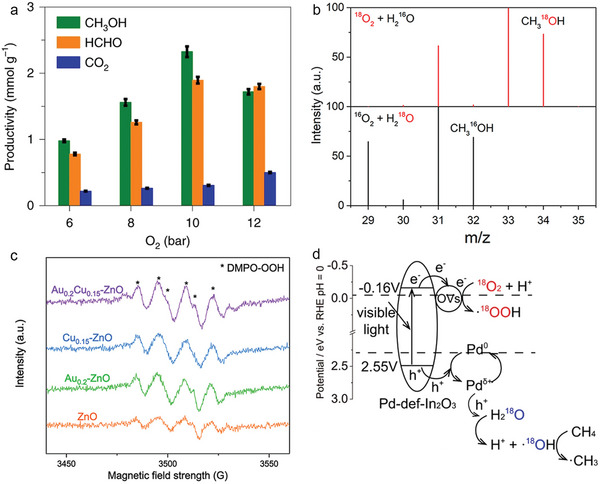
a) Productivity assessments for oxygenated products over q‐BiVO_4_ catalyst with controlled O_2_ pressure. Reproduced with permission.^[^ ^72^ ^]^ Copyright 2021, Nature Publishing Group. b) Gas chromatography‐mass spectrometry spectra of CH_3_OH generated over 0.1 wt% Au/ZnO with ^18^O_2_ + H_2_^16^O or ^16^O_2_ + H_2_^18^O as reactants. Reproduced with permission.^[^ ^67^ ^]^ Copyright 2019, American Chemical Society. c) In situ electron paramagnetic resonance spectra of •OOH over Au/Cu‐immobilized ZnO photocatalysts. Reproduced with permission.^[^ ^76^ ^]^ Copyright 2022, American Chemical Society. d) Schematic illustration of photocatalytic methane activation and oxygenate production over Pd–def–In_2_O_3_ with O_2_. Reproduced with permission.^[^ ^73^ ^]^ Copyright 2022, Nature Publishing Group.

The precise regulation of photocatalytic reaction conditions is also a necessary requirement to achieve satisfactory methane activation with suppressed overoxidation side reactions. Tang et al. reported that the formation rate of methanol and formaldehyde showed a volcanic relationship with an increase in oxygen partial pressure. Below 10 bar, the increase in oxygen partial pressure promoted the generation of reactive oxygen species. However, reduced productivity above 10 bar can be ascribed to the lower partial pressure of CH_4_ and overoxidation (**Figure** **7**a). The increase in product yield with an increasing volume of the liquid water phase is mainly due to a higher amount of dissolved CH_4_ reactant, and a dilution effect for CH_3_OH product to hinders its overoxidation. As reaction times lengthen, the selectivity for CH_3_OH decreases from 59.7% to 21.3%, while the selectivity for HCHO increases from 29.8% to 69.8%, indicating that the extension of reaction time is conducive to the further conversion of methanol to formaldehyde.^[^ ^72^ ^]^ Therefore, suitable oxygen partial pressure and reaction time with a large liquid‐phase volume may represent the ideal reaction conditions to achieve high activity and selectivity for photooxidation of methane with oxygen and water.

Isotope labelling experiments using ^13^CH_4_ as reactants are commonly employed to identify the source of carbon in the generated liquid oxygenates. In such experiments, the three distinct ^13^C nuclear magnetic resonance signals at 49, 65, and 82 ppm were attributed to CH_3_OH, CH_3_OOH, and HOCH_2_OH (methylene glycol, the major species in aqueous solutions of HCHO), respectively, confirming that all the oxygenates indeed originated from CH_4_ conversion. Isotope experiments using ^18^O‐labeled oxygen (^18^O_2_) or ^18^O‐labeled water (H_2_^18^O) as reactants were also conducted. The results indicated that all the oxygen incorporated into CH_3_OH originated from oxygen molecules rather than from water (Figure 7b).^[^ ^67^ ^]^ In addition, photogenerated reactive oxygen species can be detected by in situ electron paramagnetic resonance spectroscopy with 5,5‐dimethyl‐1‐Pyrroline N‐oxide (DMPO) as the spin‐trapping agent. For example, DMPO‐OOH and DMPO‐OH were detected over Au_0.2_Cu_0.15_‐ZnO photocatalyst under light irradiation, suggesting the generation of •OOH and •OH radicals during photocatalytic methane conversion (Figure 7c).^[^ ^76^ ^]^ Tang et al. investigated the synergistic effect of oxygen vacancies on In_2_O_3_ nanorods and Pd single atoms (Pd‐def‐In_2_O_3_).^[^ ^73^ ^]^ In this photocatalytic system, electrons were excited to the conduction band of In_2_O_3_ nanorods, while holes were left in the valence band. The photo‐excited electrons were then trapped by the oxygen vacancies, activating O_2_ with H^+^ to produced •OOH radicals. Pd atoms acted as hole acceptors (Pd + h^+^ → Pd^δ+^), which then reacted with the adsorbed H_2_O to produced •OH (Pd^δ+^ + H_2_O → Pd^0^ + •OH + H^+^). Methane was subsequently activated by the as‐produced •OH to form •CH_3_ radicals. The coupling reaction between •CH_3_ and •OOH then generated the primary products (CH_3_OOH), which was further transferred into CH_3_OH (Figure 7d).

Although the partial oxidation of methane with oxygen and water represents an ideal route for methanol production, its industrialization may still take considerable time due to the challenges such as high reaction pressures and the risk of uncontrolled excessive oxidation. Additionally, the reaction often necessitates the addition of large amount of liquid water to ensure an abundance of reactive oxygen species and reduce the concentration of methanol product, thereby inhibiting overoxidation. Consequently, the complex gas–liquid–solid interfaces between methane/oxygen reactant, water, and photocatalysts make the design of photocatalytic methane conversion systems a challenging task.

### 3.2 Methane Reforming

Methane reforming for hydrogen production mainly comprises two processes: steam reforming of methane (SRM) and dry reforming of methane (DRM), with SRM being one of the most significant methods for large‐scale hydrogen production in today's chemical industry (Equation 2).^[^ ^79^, ^80^ ^]^ SRM is an endothermic reaction between methane and water vapor, carried out at high temperatures ranging from 750 to 950 °C and high pressures of 14–20 atm. This process often leads to the generation of a substantial amount of carbon dioxide produced as a by‐product of the water–gas shift reaction (CO+H_2_O→CO_2_+H_2_).^[^ ^81^, ^82^ ^]^ DRM enables the co‐conversion of two greenhouse gases, CH_4_ and CO_2_ (Equation (3)), producing syngas as a result. This syngas severs as raw materials for the production of methanol, light olefins, and other valuable chemicals.^[^ ^83^, ^84^, ^85^ ^]^ Noble metal catalysts (such as Pt, Pd, and Ru) and non‐noble metal catalysts (mainly Ni) demonstrate excellent methane reforming performance.^[^ ^86^, ^87^, ^88^ ^]^ However, carbon deposition can occur on the catalyst surface through two processes: pyrolysis (CH_4_→2H_2_+C) and the Boudouard reaction (2CO→CO_2_+C), both leading to the catalyst deactivation.^[^ ^89^, ^90^, ^91^, ^92^ ^]^ It is worth noting that the Boudouard reaction becomes thermodynamically favorable at temperatures below 700 °C, while the pyrolysis reaction is more favorable at higher temperatures^[^ ^93^, ^94^, ^95^ ^]^

(2) $${\mathrm{CH}}_{4}+H_{2}O\to \mathrm{CO}+3H_{2},\Delta G_{298\;K}^{0}=\;142.1{\;\mathrm{kJ}\;\mathrm{mol}}^{-1}$$

(3) $${\mathrm{CH}}_{4}+{\mathrm{CO}}_{2}\to 2\mathrm{CO}+2H_{2},\Delta G_{298\;K}^{0}=\;171{\;\mathrm{kJ}\;\mathrm{mol}}^{-1}$$

The significant energy consumption involved in methane reforming leads to the re‐emission of greenhouse gases, which is contrary to the original intention of achieving sustainable development. Photocatalytic methane reforming under mild conditions is a highly anticipated alternative route that utilize renewable clean light energy as the driving force for the reaction.^[^ ^96^ ^]^ Currently, a significant portion of the research on photocatalytic methane reforming is focused on developing advanced photocatalysts with improved hydrogen or syngas production efficiency (**Table** **2**).^[^ ^97^, ^98^ ^]^

**Table 2:** Representative studies on the photocatalytic methane reforming.

Catalyst	Reactants	Reaction conditions	Performance	Ref.
Ru/TiO_2_‐H_2_	8 vol% CO_2_, 8 vol% CH_4_, 84 vol% Ar 50 mL min^−1^	5.0 mg catalyst, 300 W Xe lamp focused by an achromatic lens, 12.0 W cm^−2^, 441°C	CO: 708.4 mmol g_cat_^−1^ h^−1^ H_2_: 645.5 mmol g_cat_^−1^ h^−1^	[99]
Rh/SrTiO_3_	1 vol% CO_2_, 1 vol% CH_4_, 98 vol% Ar 10 mL min^−1^	5.0 mg catalyst, 150 W Hg–Xe lamp, 300°C	CO: ≈4.5 µmol min^−1^, H_2_: ≈4.5 µmol min^−1^	[100]
1% Rh/LaNiO_3_	50 vol% CO_2_, 50 vol% CH_4_, batch,1 h	50 mg catalyst, 300 W Xe lamp 1.5 W cm^−2^, 280 °C	H_2_:452.3 mmol g_Rh_^−1^ h^−1^, CO:527.6 mmol g_Rh_^−1^ h^−1^	[101]
Rh/Ce_x_WO_3_	20% CH_4_, 20% CO_2_, 60% Ar, 50 mL min^−1^	50 mg catalyst, 300 W Xe lamp (300–1100 nm), 1.8 W cm^−2^	CO: 152.3 mmol g_Rh_^−1^ h^−1^, H_2_: 88.5 mmol g_Rh_^−1^ h^−1^	[102]
SCM‐Ni/SiO_2_	11.7 vol% CH_4_ 11.5 vol% CO_2_, 118.7 mL min^−1^	0.0243 g catalyst, 500 W Xe lamp	H_2_: 17.1 mmol min^−1^ g^−1^ CO: 19.9 mmol min^−1^ g^−1^	[103]
Rh/TiO_2_‐B	1% CH_4_ 1% CO_2_ 98% Ar 10 mL min^−1^	8.6 mg catalyst, 150 W Hg‐Xe lamp	H_2_: 21.5 mmol h^−1^ g^−1^ CO: 21.2 mmol h^−1^ g^−1^	[104]
Pt‐K_2_Ti_6_O_13_	1.5% CH_4_ 50% H_2_O 40 mL min^−1^	0.8 g catalyst, 300 W Xe lamp	H_2_: 0.8 µmol min^−1^ CO_2_: 0.2 µmol min^−1^	[105]
La‐NaTaO_3_	25% CH_4_ 2.4% H_2_O 72.6% Ar 15 mL min^−1^	1.2 g catalyst, 254 ± 10 nm light, 165 mW cm^−2^	H_2_: 21 µmol h^−1^ CO_2_: 4 µmol h^−1^	[106]
2% Rh/TiO_2_	10% CH_4_, 3% H_2_O vapor 10 mL min^−1^	20 mg catalyst, LA‐251 Xe lamp, 260 °C	H_2_: ≈116 µmol g^−1^ min^−1^	[107]

**Figure 8:**
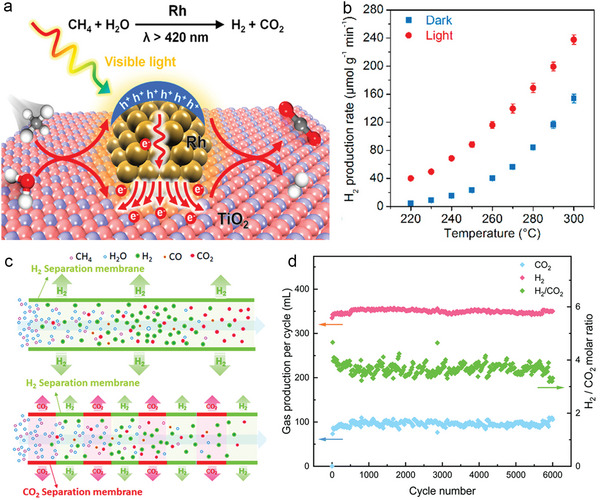
a) Photocatalytic SRM reaction on Rh/TiO_2_ under visible light illumination. b) H_2_ production rates over Rh/TiO_2_ photocatalyst as a function of reaction temperature under light or dark conditions. a,b) Reproduced with permission.^[^ ^107^ ^]^ Copyright 2018, American Chemical Society. c) Schematic illustration of H_2_ single separation and sequential separation of H_2_ and CO_2_. d) H_2_/CO_2_ production volume and ratio over 6000 cycles of SRM reaction at an average CH_4_ conversion of 95.41%. c,d) Reproduced with permission.^[^ ^110^ ^]^ Copyright 2022, The Royal Society of Chemistry.

Transition metal oxide‐based photocatalysts are widely utilized for methane reforming due to their abundant surface acid‐base sites, redox properties, defect structures and the ability to generate reactive oxygen species.^[^ ^108^, ^109^ ^]^ TiO_2_‐supported Rh nanoparticles have been successfully applied to photocatalytic SRM with excellent performance at mild operating temperatures below 300°C (**Figure** **8**a).^[^ ^107^ ^]^ In this process, the photogenerated hot electrons and holes in the metal nanoparticles can efficiently transfer to electron acceptors and donors, respectively. For instance, hot electrons can be transferred to the conduction band of the semiconductor through the Schottky barrier at the metal‐semiconductor interfaces, effectively extending their lifetime. The H_2_ production rate was consistently higher under light conditions compared to dark conditions, and the photocatalytic SRM system reduces the apparent activation energy by about 50% compared to the thermocatalytic route (Figure 8b). In addition, both Rh/ZrO_2_ and Rh/SiO_2_ showed much less improvements in reaction activities than that of Rh/TiO_2_ under light conditions. Transient absorption spectra revealed an ultrafast separation of hot carriers at the Rh‐TiO_2_ interface, which creates abundant electron‐deficient Rh^δ+^ species as active sites to promote methane activation at low temperatures.

High reaction temperature (>800 °C), process complexity, and challenging separation of H_2_ and CO_2_ have been significant limitations for SRM. Hao et al. introduced a novel concept of multiproduct sequential separation and a new method of sequential separation‐driven SRM (Figure 8c).^[^ ^110^ ^]^ Unlike the conventional “reformer‐heat exchanger‐shift reactor‐H_2_ purifier” layout used in industrial SRM, their reactor incorporates nickel SRM catalyst and hydrotalcite CO_2_ sorbent arranged alternately to achieve sequential separation of H_2_ and CO. This innovative setup enables the direct obtention of high‐purity H_2_ and CO_2_ with >99% conversion of methane and >99% yield and selectivity under 400 °C and 1 bar. When exposed to direct solar irradiation, the solar energy efficiency for H_2_ production reaches 3.4% (Figure 8d). By leveraging advancement in solar thermal energy storage technology, medium and low‐temperature SRM catalysts, and CO_2_ adsorbent technology, it is possible to increase the efficiency to 46.5% or higher. Furthermore, the incorporation of low‐energy‐penalty separation technologies can potentially elevate the efficiency to over 60%.

While methane steam reforming represents a well‐established industrial production method, it continues to rely on high temperatures ranging from 800 to 1000 °C to facilitate the endothermic methane reforming process. This not only leads to substantial energy consumption but also diminishes the catalyst's operational lifespan. In the realm of catalyst research, the pursuit of advanced catalysts exhibiting heightened reactivity and stability remains a primary objective within methane steam reforming studies. Such advancements hold the potential to lower reaction temperatures and reduce catalyst costs. On the other hand, traditional methane steam reforming predominantly employs thermal catalysis, entailing significant consumption of fossil fuels and raising concerns regarding greenhouse gas emissions. Shifting toward methane steam reforming driven by clean and sustainable solar energy has the potential to usher in a transformative industrial revolution within the existing industrial framework.

**Figure 9:**
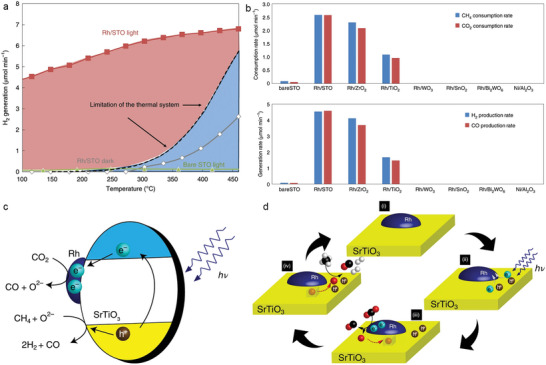
a) The temperature dependence of DRM activity over Rh/STO in light and dark conditions. Dashed line represents the limitation of the thermal DRM system using a generic catalyst. b) The photocatalytic consumption rates of CH_4_ and CO_2_ and the production rates of H_2_ and CO for Rh/STO and other Rh‐loaded metal oxides. c) Schematic illustration of the migration of photo‐excited charge carriers with CH_4_ and CO_2_ over Rh/STO. d) Reaction paths for photocatalytic DRM over Rh/STO. Reproduced with permission.^[^ ^100^ ^]^ Copyright 2020, Nature Publishing Group.

For DRM reactions, SrTiO_3_‐supported Rh (Rh/STO) catalyst has been reported to enhance the reaction efficiency under ultraviolet irradiation at low temperatures (300°C), breaking the theoretical limitations of conventional thermocatalytic systems (**Figure** **9**a).^[^ ^100^ ^]^ Rh/STO achieves a high CH_4_ conversion rate of about 57% with a 5.9% quantum efficiency. The production rates of H_2_ and CO are both around 2.5 µmol min^−1^, while only trace amounts of products are detected over bare STO (Figure 9b). A lattice oxygen‐mediated photocatalytic mechanism is proposed for the activation and conversion of CH_4_ and CO_2_ molecules. Photogenerated electrons are transferred from the conduction band of STO to the surface of Rh particles, thereby reducing CO_2_ to produce CO and the remaining O^2−^ ions are introduced into the STO lattice. The photogenerated holes in the STO valence band can activate lattice oxygen and then methane to produce CO and H_2_. This lattice oxygen‐mediated photocatalytic mechanism provides a useful guideline for the design of transition metal oxide‐based catalysts in methane reforming reactions (Figure 9c,d).

**Figure 10:**
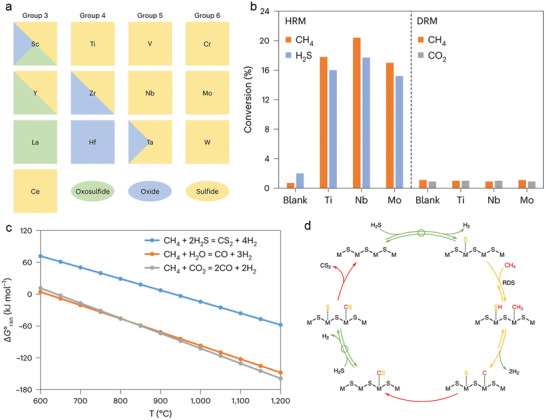
a) Bulk phase composition of spent catalysts derived from metal oxides of groups 3–6 after HRM reaction characterized by X‐ray diffraction. b) CH_4_ conversion over catalysts derived from Ti, Nb and Mo‐based metal oxides for DRM and HRM reactions. c) Gibbs free energies of different methane‐reforming reactions as a function of temperature calculated based on values of the NIST (National Institute of Standards and Technology) database. d) Scheme of a HRM cycle on metal sulfides mediated by sulfur adatoms. a–d) Reproduced with permission.^[^ ^111^ ^]^ Copyright 2023, Nature Publishing Group.

In addition to the intensively studied photocatalytic SRM and DRM reactions, H_2_S reforming of methane (HRM), using H_2_S as a CH_4_ reforming coreactant, has shown promising potential for combining acidic natural gas purification with large‐scale H_2_ production. In this reaction, the by‐product CS_2_ plays a crucial role in synthesizing various and sulfur‐containing organic chemicals. Johannes A. Lercher and co‐workers reported that metal oxides of group 4 to 6 elements are unreactive catalysts in steam and dry reforming reactions, but they high activity and stability as (pre)catalysts for the HRM reaction.^[^ ^111^ ^]^ Under reaction conditions (900 °C, 2 h), most metal oxides undergo partial or complete conversion to their corresponding metal sulfides, with group 5 and 6 metal oxides showing complete sulfidation (**Figure** **10**a).

Catalysts derived from Ti, Nb and Mo‐based metal oxides were found to be inactive for the DRM at 900 °C, but they exhibited significant catalytic activity for HRM (Figure 10b). Given that HRM is thermodynamically more demanding than SRM and DRM (Figure 10c), these results hint that relatively clean (that is, no steady‐state surface S* coverages) surfaces of metal oxides and sulfides are unable to activate methane at these conditions. During the reaction, the sulfur species are dynamically bound to metal cations and act as the key active sites. The H_2_S decomposition and surface hydrogen recombination steps are in quasi‐equilibrium, while the CH_4_ dissociation step is reversible but not in quasi‐equilibrium. S*‐mediated C–H bond breaking is the possible rate‐determining step for all catalysts. Additionally, the 3d and 4d/5d catalysts exhibit subtle but essential differences in the thermodynamic stability of S* (Figure 10d).

### 3.3 Methane Coupling

Methane coupling to C_2+_ hydrocarbons (mainly ethane and ethylene) offers another avenue for directly converting methane into valuable chemicals.^[^ ^112^, ^113^ ^]^ This process is categorized into two types based on the presence or absence of oxidizing agents (Equations (4)–(7)): oxidative coupling of methane (OCM) and nonoxidative coupling of methane (NOCM)

(4) $$2{\mathrm{CH}}_{4}\to C_{2}H_{6}+H_{2},\Delta G_{298\;K}^{0}=\;68.8{\;\mathrm{kJ}\;\mathrm{mol}}^{-1}$$

(5) $$2{\mathrm{CH}}_{4}\to C_{2}H_{4}+2H_{2},\Delta G_{298\;K}^{0}=\;169.6{\;\mathrm{kJ}\;\mathrm{mol}}^{-1}$$

(6) $$4{\mathrm{CH}}_{4}+O_{2}\to 2C_{2}H_{6}+2H_{2}O,\Delta G_{298\;K}^{0}=\;-320{\;\mathrm{kJ}\;\mathrm{mol}}^{-1}$$

(7) $$2{\mathrm{CH}}_{4}+O_{2}\to C_{2}H_{4}+2H_{2}O,\Delta G_{298\;K}^{0}=\;-288{\;\mathrm{kJ}\;\mathrm{mol}}^{-1}$$

Due to thermodynamic limitations, NOCM often requires very high reaction temperatures to achieve acceptable methane conversion efficiency. For example, Bale et al. demonstrated a NOCM process using single iron sites embedded in a silica matrix, achieving a maximum methane conversion of 48.1% and ethylene selectivity of 48.4% through NOCM at high temperatures up to 1363 K.^[^ ^114^ ^]^ But such high‐temperature operation often results in carbon deposition on the catalyst, leading to catalyst deactivation and reducing its lifetime. This challenge has hindered the practical industrial application of the catalytic system.^[^ ^115^, ^116^ ^]^ With the introduction of oxidants, primarily O_2_, the OCM process can coupling methane into light olefins under favorable thermodynamic conditions. In 1982, Keller and Bhasin first proposed the OCM process to produce ethylene and other raw chemicals, which marked a significant milestone in methane conversion research and garnered widespread attention.^[^ ^117^ ^]^ However, the presence of oxygen in the reaction system inevitably leads to the generation of by‐products such as CO and CO_2_.^[^ ^118^, ^119^, ^120^ ^]^

To date, extensive efforts have been dedicated to the development of high‐performance catalysts for methane coupling, accompanied by thorough investigations into reaction mechanisms and rector optimizations, resulting in notable progress in this field.^[^ ^121^, ^122^, ^123^ ^]^ Among these catalysts, Na_2_WO_4_/SiO_2_ has been recognized as one of the most promising catalysts, although thermocatalytic OCM still requires high temperatures of up to 750 °C.^[^ ^124^, ^125^, ^126^ ^]^ In recent years, research on photocatalytic methane coupling under mild conditions based on transition metal oxides (ZnO, TiO_2_, Nb_2_O_5_, WO_3_, etc.) has emerged as a prominent area of focus and has rapidly become a hot topic in the field of methane conversion (**Table** **3**).^[^ ^127^, ^128^, ^129^, ^130^, ^131^, ^132^, ^133^ ^]^

**Table 3:** Representative studies on the photocatalytic methane coupling.

Catalyst	Reactants	Reaction conditions	Performance	Ref.
Pd_1_/TiO_2_	1338 µmol CH_4_	3.0 mg catalyst, 300 W Xe lamp, 600 mW cm^−2^	C_2_H_6_: 0.91 mmol g^−1^ h^−1^	[134]
ZnO‐AuPd_2.7%_	22.3 µmol CH_4_	2 mg catalyst, 300 W Xe lamp, 600 mW cm^−2^, 4 h	C_2_H_4_: 105.5 nmol C_2_H_6_: 199.7 nmol C_3_H_6_: 4.4 nmol C_3_H_8_: 5.4 nmol CO: 21.6 nmol	[40]
873K‐Nb_2_O_5_	45 mL CH_4_	5 mg catalyst, 300 W Xe lamp, 4h	C_2_H_6_: 600.8 µmol g^−1^ h^−1^ C_3_H_8_: 62.3 µmol g^−1^ h^−1^ C_4_H_10_: 17.0 µmol g^−^h^−1^ H_2_: 780.9 µmol g^−1^ h^−1^	[133]
Pt/HGTS (2%)	44.6 µmol CH_4_	0.2 g catalyst, 300 W Xe lamp, 4 h;	H_2_: ≈7 µmol C_2_H_6_: ≈4 µmol C_3_H_6_: ≈1.4 µmol	[128]
Ga_0.75_Zn_0.25_N_0.75_O_0.25_	300 µmol CH_4_	2 mg catalyst, 300 W Xe lamp, 2 h	CH_4_ conversion: 331 µmol g^−1^ h^−1^ C_2_H_6_ selectivity: 98%	[129]
Pd(0.1)/Ga_2_O_3_	10% CH_4_ in Ar 30 mL min^−1^	0.8 g catalyst, ceramic xenon lamp (254 ± 10 nm), 20 mW cm^−2^	H_2_: 1.2 µmol h^−1^ C_2_H_6_: 0.51 µmol h^−1^	[135]
Ag‐HPW/TiO_2_	CH_4_ and Air	0.1 g catalyst, 400 W Xe lamp	C_2_H_6_: ≈20.6 µmol g^−1^ h^−1^ hydrocarbons selectivity: 90%	[136]
Cu_0.1_Pt_0.5_/PC‐50	10% CH_4_, O_2_:CH_4_ = 1:400 GHSV = 24 000 g^−1^ h^−1^	0.1 g catalyst, 40 W 365 nm LED	C_2_H_6_ and C_2_H_4_: 6.8 µmol h^−1^	[137]
Au‐ZnO/TiO_2_	69 mL min^−1^ CH_4_, 1 mL min^−1^ air	20 mg catalyst, 300 W Xe lamp (300–500 nm), 500 mW cm^−2^	C_2_H_6_: 5020 µmol g^−1^ h^−1^ C_2_H_4_: 20 µmol g^−1^ h^−1^ C_3_H_8_: 225 µmol g^−1^ h^−1^ CO: 16 µmol g^−1^ h^−1^ CO_2_: 278 µmol g^−1^ h^−1^	[138]
Au/ZnO	O_2_:CH_4_ = 1:99	5 mg catalyst,100 W 365 nm LED, 350 mW cm^−2^	C_2_–C_4_: 683 µmol g^−1^ h^−1^, C_2_–C_4_ selectivity: 83%	[139]
Ag‐AgBr/TiO_2_	40 mL min^−1^ CH_4_, 1 mL min^−1^ Air, 360 mL min^−1^ Ar, 6 bar	100 mg catalyst, 365 nm LED, 40°C	C_2_H_6_: 35.4 µmol h^−1^, C_2_H_6_ selectivity: 79%	[140]
TiO_2_(Si)‐Cu	CH_4_, 5 mL H_2_O	5 mg catalyst, 300 W Xe lamp, 200 mW cm^−2^,4 h	C_2_H_6_: 33.8 µmol g^−1^ h^−1^, C_2_H_6_ selectivity: 88.4%	[141]

**Figure 11:**
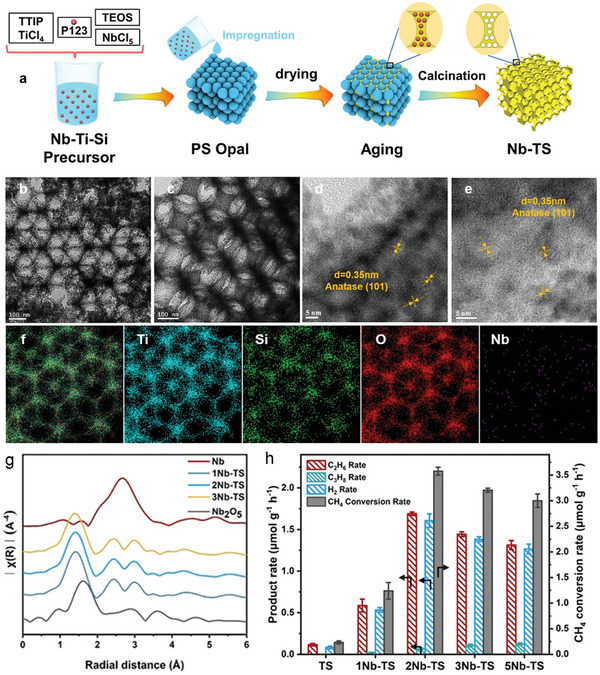
a) Scheme of the preparation of Nb‐TS photocatalysts. TEM images of b) TS and c) 2Nb‐TS. High‐resolution TEM images of d) TS and (e) 2Nb‐TS. f) Elemental mapping of Ti, Si, O, and Nb for 2Nb‐TS. g) Nb K‐edge extended X‐ray absorption fine structure of Nb‐TS and reference samples. h) Production rates of hydrocarbons and hydrogen, and methane conversion rates over Nb‐TS photocatalysts. Reproduced with permission.^[^ ^142^ ^]^ Copyright 2021, Wiley‐VCH.

Zhang et al. developed a novel catalyst by doping single‐atom Nb into hierarchical porous TiO_2_–SiO_2_ (Nb‐TS) microarray, using hexagonally arranged ordered polystyrene (PS) opals as hard templates (**Figure** **11**a).^[^ ^142^ ^]^ The research team then systematically investigated the role of surface‐localized electrons induced by the n‐type dopants in the methane activation and ethane desorption processes in photocatalytic NOCM. The unique hierarchical porous structure of the photocatalyst exhibits interconnected pores and high specific surface area, enabling highly dispersed doping of carious transition metal ions (Figure 11b–f). In this case, Nb is automatically dispersed in the lattice of TS with a Nb–O coordination structure (Figure 11g). This arrangement not only facilitates the separation of photogenerated charge carriers but also serves as an active site for methane activation, resulting in a remarkable methane conversion rate of 3.57 mmol g−^1^ h^−1^ and excellent cycling stability for in photocatalytic NOCM (Figure 11h).

**Figure 12:**
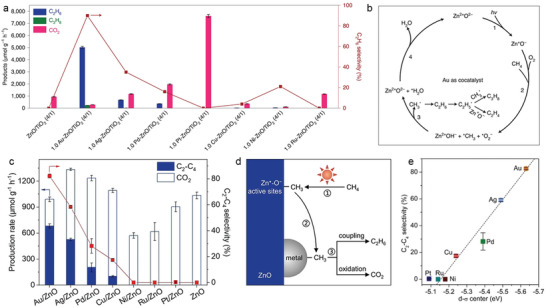
a) Photocatalytic OCM activity over ZnO/TiO_2_ loaded with different metal cocatalysts. b) Proposed reaction processes on ZnO loaded with Au cocatalyst. a,b) Reproduced with permission.^[^ ^138^ ^]^ Copyright 2021, Nature Publishing Group. c) Photocatalytic OCM activity over ZnO and different metal/ZnO samples. d) Scheme of the proposed photocatalytic OCM mechanism on metal‐loaded ZnO. e) Calculated linear relationship between d‐σ center and C_2+_ product selectivity over metal/ZnO photocatalysts. c–e) Reproduced with permission.^[^ ^139^ ^]^ Copyright 2023, Wiley‐VCH.

Thermocatalytic OCM is generally believed to follow the heterogeneous‐homogeneous catalytic reaction mechanism, where methane activation to methyl radicals takes place on the catalyst surface, and the subsequent coupling of methyl radicals is a spontaneous homogeneous reaction in the gas phase. This implies that catalyst engineering along may have limited control over OCM selectivity.^[^ ^143^, ^144^, ^145^ ^]^ Regulating the interaction between methyl species and catalyst through a photocatalytic route could be an important approach to enhance the product selectivity. Ye et al. reported the use of Au nanoparticle‐loaded ZnO/TiO_2_ hybrid in photocatalytic OCM, showing an ethane production rate of over 5000 µmol g^−1^ h^−1^ with 90% selectivity (**Figure** **12**a).^[^ ^138^ ^]^ The presence of Au cocatalyst promoted the desorption of methyl adsorbates from the ZnO/TiO_2_ surface, leading to the formation of methyl radicals in the gas phase. This process inhibits the excessive oxidation of methyl groups and facilitates the formation of ethane. In contrast, Pt, which possesses a strong oxygen reduction capacity, promotes the binding of adsorbed methyl and oxygen species, leading to the generation of abundant *OCH_3_. These *OCH_3_ species are considered to be key intermediates in the excessive oxidation process, ultimately resulting in the production of CO_2_ (Figure 12b). Zhang et al. discovered that ZnO photocatalysts modified with various metal nanoparticles (metal/ZnO) exhibit different selectivity in coupling product during photocatalytic OCM (Figure 12c).^[^ ^139^ ^]^ Photogenerated reactive oxygen species on ZnO activate methane to form methyl adsorbates. Subsequently, the adsorbed methyl species migrate to the metal surface and undergo either coupling to ethane or overoxidization to carbon dioxide (Figure 12d), depending on the d‐σ hybridization effect associated with methyl‐metal interfacial interactions (Figure 12e).

### 3.4 Methane Combustion

Due to its high H/C ratio, methane is a cleaner fuel alternative to coal and oil with fewer CO_2_ emissions. However, the residual methane in the tailpipe, which is not sufficiently reacted, can have an unpleasant greenhouse effect approximately 20 times stronger than that of CO_2_.^[^ ^146^, ^147^ ^]^ To address this issue, the methane combustion reaction (Equation (8)), representing the total oxidation of methane, has become a research focus for the removal of residual methane.^[^ ^148^ ^]^ In the direct combustion process, the entire reaction follows the free radical chain reaction mechanism once the ignition temperature is reached. The reaction temperatures quickly soar to as high as 1200 °C.^[^ ^149^ ^]^ However, higher temperatures increase the possibility of methane incomplete combustion, leading to the emission of carbon monoxide, carbon smoke particles, and other hydrocarbon byproducts. nitrogen in the air can be oxidized to toxic nitrogen oxides during the combustion process. Compared with direct combustion, catalytic combustion offers better control over the reaction process and allows for lower reaction temperatures, usually below 1000 °C, resulting in reduced emission of carbon contaminants and nitrogen oxides.^[^ ^150^, ^151^ ^]^ Given that thermocatalytic methane combustion still requires relatively high temperatures (>400°C), photocatalytic methane combustion as a branch of environmental photocatalysis has emerged as a promising alternative pathway that operates at low temperatures^[^ ^152^, ^153^ ^]^

(8) $${\mathrm{CH}}_{4}+2O_{2}\to {\mathrm{CO}}_{2}+2H_{2}O,\Delta G_{298\;K}^{0}=\;-801{\;\mathrm{kJ}\;\mathrm{mol}}^{-1}$$

**Figure 13:**
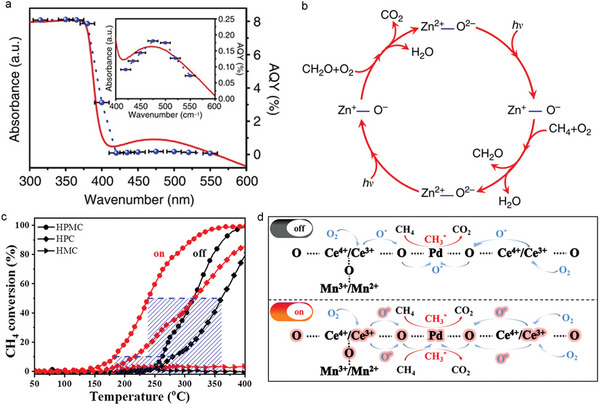
a) Ultraviolet‐visible diffuse reflectance spectrum and wavelength‐dependent apparent quantum yield of Ag/ZnO. b) Schematic illustration of the photocatalytic CH_4_ combustion processes over Ag/ZnO. a‐b) Reproduced with permission.^[^ ^155^ ^]^ Copyright 2016, Nature Publishing Group. c) CH_4_ conversion curves over HPMC and control samples with/without irradiation. HPC: PdO/CeO_2_ coated on halloysite nanotubes, HMC: Mn_3_O_4_/CeO_2_ coated on halloysite nanotubes. d) Proposed photocatalytic CH_4_ combustion mechanism over HPMC. a‐d) Reproduced with permission.^[^ ^156^ ^]^ Copyright 2021, Wiley‐VCH.

Reactive oxygen species produced by transition metal oxides under light irradiation play a crucial role in various photocatalytic environmental applications.^[^ ^154^ ^]^ Ag/ZnO nanoparticles, for example, demonstrate highly effective photocatalytic oxidation of methane via surface plasmon resonance under simulated sunlight.^[^ ^155^ ^]^ The quantum yield of this Ag/ZnO nanomaterial can reach as high as 8% under ultraviolet irradiation (<400 nm), and it remains above 0.1% at 470 nm (**Figure** **13**a). The photogenerated Zn^+^–O− on ZnO are believed to act as the active site for methane combustion (Figure 13b). In addition, the Ag/ZnO photocatalyst also demonstrate excellent photocatalytic combustion performance for other hydrocarbons, such as ethane, propane, and ethylene, which holds promising potential for the photocatalytic purification of volatile organic compound in the atmosphere.

Zhang et al. reported the synthesis of a PdO/Mn_3_O_4_/CeO_2_ (HPMC) photocatalyst that self‐assembled into a compact coating on the surface of halloysite nanotubes by triggering the autooxidation–reduction reaction of reductive Ce(OH)_3_ and oxidative MnO_4_^−^/Pd^2+^ ions in water.^[^ ^156^ ^]^ The PdO, Mn_3_O_4_, and CeO_2_ components exhibit a strong synergic effect, resulting in a significantly reduced CH_4_ ignition temperature to 180°C under visible light irradiation (Figure 13c). Additionally, the apparent activation energy is reduced from 206.4 kJ mol^−1^ in the dark condition to 75.6 kJ mol^−1^ in the light condition. According to the classical Mv‐K mechanism, the rate‐determining step of CH_4_ conversion is the activation of oxygen molecules to form reactive oxygen species. Therefore, the increase in the photocatalytic activity of HPMC can be attributed to the significant increase in the concentration of Ce^3+^, which promotes the rapid and stable redox equilibrium of PdO→Pd→PdO during the oxygen activation process (Figure 13d).

### 3.5 Photocatalytic Methane Functionalization

**Figure 14:**
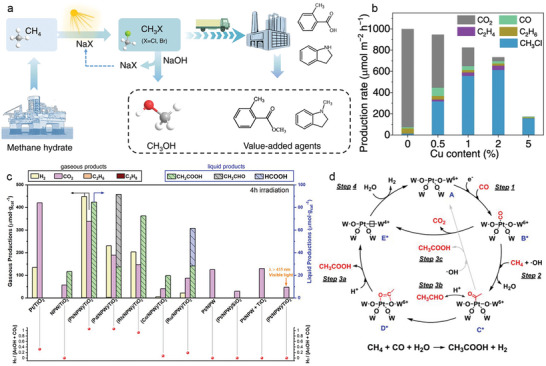
a) Photocatalytic methane halogenation using alkali halide as a halogenation agent, and the successive transformations into fuels and chemicals. b) Photocatalytic production rates of CH_3_Cl and by‐products over Cu–TiO_2_. Reproduced with permission.^[^ ^163^ ^]^ Copyright 2023, Nature Publishing Group. c) Photocatalytic CH_3_COOH production activities of (Pt/NPW)/TiO_2_ and control samples with methane, carbon monoxide and water as reactants. d) Proposed mechanism of the photocatalytic CH_3_COOH generation process over a 4‐fold oxygen coordinated Pt single atom site. Note that from states B to E, the * marks the reduction state of NPW due to accepting photoexcited electrons from TiO_2_. c,d) Reproduced with permission.^[^ ^164^ ^]^ Copyright 2023, American Chemical Society.

Selective catalytic functional group reactions of C–H bonds are favorable methods for constructing C–C and C–X bonds, especially in the synthesis of drug molecules.^[^ ^157^, ^158^, ^159^ ^]^ The introduction of methyl groups can significantly enhance the bioactivity of the molecule, a phenomenon known as the “magic methyl effect” in biology, providing a simple and effective approach for the development of active drug molecules.^[^ ^160^ ^]^ Besides, methane is considered a promising methylation reagent to replace the toxic dimethyl sulphate and iodomethane commonly used at present.^[^ ^161^, ^162^ ^]^ The Cu‐doped TiO_2_ photocatalyst efficiently activates methane molecules and alkali metal halides when exposed to full spectrum or visible light, enabling an efficient methane halogenation reaction for the preparation of halogenated hydrocarbons.^[^ ^163^ ^]^ Moreover, tandem reactions have demonstrated that the halogenated products can be further transferred to methanol and drug intermediates (**Figure** **14**a). This approach affords a methyl halide production rate of up to 0.61 mmol h^−1^ m^−2^ for chloromethane (Figure 14b).

In another study, Vitaly V. Ordomsky et al. reported a novel photocatalytic system consisting of TiO_2_‐supported ammonium phosphotungstic polyoxometalate clusters anchored with isolated Pt single atoms [(Pt/NPW)/TiO_2_], where CH_4_, CO, and H_2_O are directly converted to acetic acid with a selectivity over 90% and 66% to acetic acid on liquid‐phase and carbon basis (Figure 14c).^[^ ^164^ ^]^ In this process, CH_4_ is activated by photogenerated •OH radicals and reacts with CO molecules adsorbed on the surface Pt single atoms to form acetyl groups, which is further oxidized to produce acetic acid (Figure 14d).

**Figure 15:**
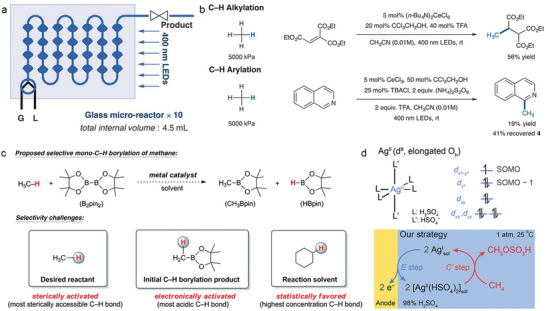
a) Photocatalytic amination of alkanes in continuous‐flow photoreactors for scaled‐up applications. b) Photocatalytic alkylation and arylation reactions with methane. a,b) Reproduced with permission.^[^ ^165^ ^]^ Copyright 2018, American Association for the Advancement of Science. c) Reactivity and selectivity challenges in the C–H borylation of methane. Reproduced with permission.^[^ ^166^ ^]^ Copyright 2016, American Association for the Advancement of Science. d) The frontier orbitals and structure of a proposed Ag^II^ metalloradical in 98% H_2_SO_4_ and the proposed electrocatalytic CH_4_ activation strategy. SOMO, Singly Occupied Molecular Orbital. Reproduced with permission.^[^ ^167^ ^]^ Copyright 2021, Wiley‐VCH.

In addition to the transition metal oxide‐based photocatalysts, homogeneous photocatalysts also demonstrates excellent photocatalytic methane conversion performance, especially on methane functionalization. Zuo et al. reported the photocatalytic selective amination of methane at room temperature, utilizing the synergistic catalysis of cerium trifluoromesylate and trichloroethanol, obtaining a turnover number (TON) of up to 2900 (**Figure** **15**a).^[^ ^165^ ^]^ Ethane, another major component of natural gas, can also be selectively converted to amines with a TON up to 9700. Furthermore, this photocatalytic system allows for a series of methane functionalization reactions, such as alkylation and arylation of methane, with high efficiency and selectivity (Figure 15b). Notably, the photocatalyst can regulate the electrophilicity of the alkoxy radical by altering the structure of the alcohol compound as the hydrogen transfer catalyst, enabling regional selective regulation of multiple types of C–H bonds, including the two different substitution types of C–H bonds in propane and butane molecules. In addition, the gas–liquid two‐phase flow photochemical reaction involving methane, ethane and other gases has been successfully realized through flow chemistry and glass microreactor technology, yielding satisfactory conversion efficiency, laying a foundation for the scale‐up application.

Sanford et al. employed soluble iridium, rhodium and ruthenium complexes as catalysts to complete the methane borylation reaction at 150 °C and 2800–3500 kPa in cyclohexane solvent by adding bis‐pinacolborane to methane (Figure 15c).^[^ ^166^ ^]^ Liu et al. demonstrated that continuous generation of divalent silver metal radicals in concentrated sulfuric acid through the electrochemical oxidation of mono‐valent silver metal precursors allows the functionalization of methane into methyl bisulfate (CH_3_OSO_3_H) at room temperature and atmospheric pressure (Figure 15d).^[^ ^167^ ^]^

## 4 Advanced Reactor Design

In addition to the development of advanced photocatalysts, designing efficient photocatalytic reactors is also a key focus in photocatalytic methane conversion. Photoreactors are more complex than traditional thermocatalytic reactors, requiring consideration of various factors. Except reactant/product mass transfer, flow mode, catalyst loading, and temperature control, photoreactors also require careful consideration of light source configuration, such as light propagation and distribution, light uniformity and permeability, and the characteristics of the light source (intensity, frequency, monochromaticity, polarization, etc.). These considerations pose challenges for both laboratory research and industrial application, especially for heterogeneous photocatalyst system.

Research on advanced photoreactors primarily focus on regulating the transfer process of photons to the light harvesting unit and optimizing the mass transfer process of reactants to the active site of photocatalysts. In other words, efforts are made to improve the efficiency of light and mass transfer within the photoreactor.

**Figure 16:**
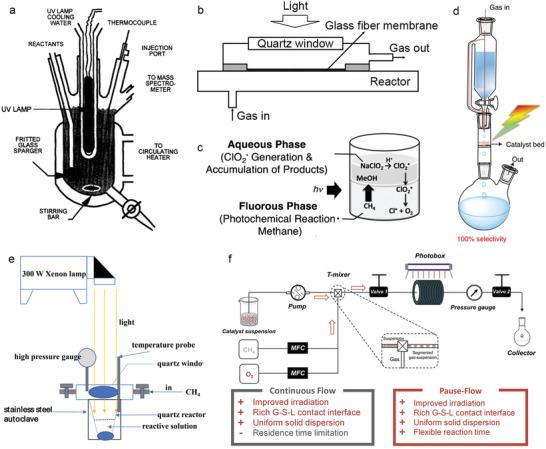
a) Schematic illustration of a classical methane conversion photoreactor. Reproduced with permission.^[^ ^168^ ^]^ Copyright 1997, Elsevier. b) The home‐made flow photocatalytic OCM reactor made from stainless‐steel equipped with a quartz window on its top. Reproduced with permission.^[^ ^137^ ^]^ Copyright 2020, Wiley‐VCH. c) Two‐phase photooxidation of CH_4_ with NaClO_2_. CH_4_ is dissolved in the fluorous phase and NaClO_2_ is dissolved in the aqueous phase. The oxygenated products are accumulated in the aqueous phase. Reproduced with permission.^[^ ^169^ ^]^ Copyright 2018, Wiley‐VCH. d) Scheme of the continuous‐flow apparatus for methane‐to‐methanol photoconversion. Reproduced with permission.^[^ ^170^ ^]^ Copyright 2022, Nature Publishing Group. e) Typical high‐pressure photoreactor used for photocatalytic methane oxidation. Reproduced with permission.^[^ ^171^ ^]^ Copyright 2020, The Royal Society of Chemistry. f) Scheme of the “pause‐flow” photoreactor. MFC: mass flow controller. Reproduced with permission.^[^ ^172^ ^]^ Copyright 2022, American Chemical Society.

The simple quartz photochemical reaction vessel, equipped with a Teflon‐coated magnetic stirring bar, a fritted glass sparger, a nitrogen line used to cool the light source, and an injection port, has been a long been long‐standing choice for photocatalytic methane conversion experiments (**Figure** **16**a).^[^ ^168^ ^]^ For photocatalytic methane oxidative coupling, a home‐made stainless steel flow reactor with a quartz window and a glass fiber membrane holding uniformly dispersed photocatalyst has been used (Figure 16b).^[^ ^137^ ^]^ Two‐phase perfluorohexane/H_2_O systems have been used to achieve methane photooxidation to produce oxygenated products like CH_3_OH and HCOOH under ambient conditions (Figure 16c).^[^ ^169^ ^]^ In this system, methane oxygenation is initiated by the photochemical Cl–O bond cleavage of ClO_2_• to generate Cl• and O_2_. The produced Cl• reacts with CH_4_ to form a methyl radical, and the use of a fluorous solvent successfully prolongs the lifetime of the reactive radical species.

A flow of CH_4_/O_2_‐saturated water (15 mL h^−1^) is passed through a packed layer of metal‐organic‐framework immobilized with mono‐iron hydroxyl sites [PMOF‐RuFe(OH)], achieving 100% selectivity for methanol production with a production rate of 8.81 ± 0.34 mmol g_cat_^−1^ h^−1^, surpassing the activity of methane monooxygenase (Figure 16d).^[^ ^170^ ^]^ To achieve room temperature methane conversion in aqueous solution, high pressure is often required to achieve considerable spatiotemporal yields due to methane's low solubility in water. High‐pressure stainless steel autoclave has been extensively used in the selective conversion of CH_4_ to CH_3_OH, demonstrating excellent photocatalytic performance (Figure 16e).^[^ ^171^ ^]^ Tang et al. reported a novel flow photocatalytic reactor consisting of a narrow optical path for uniform irradiation and a backpressure valve to increase methane pressure, showing possibility for scaling up the photocatalytic methane‐to‐formaldehyde conversion.^[^ ^172^ ^]^ A well‐designed “pause‐flow” microtube reactor showed unique advantages in flexible control over the reaction time and sufficient mixing of reactants and photocatalysts (Figure 16f). This design enabled efficient, selective, continuous, and stable production of formaldehyde via photocatalytic methane oxidation under mild conditions.

## 5 Summary and Outlook

Methane is not only a clean fuel but also a promising chemical raw material, making methane catalytic conversion a prominent research area. Starting from methane, various valuable products can be obtained through heterogeneous catalysis, such as syngas through stream and dry reforming, methanol, C_2+_ alkane/olefins, and even aromatic hydrocarbons via partial oxidation, coupling, and other reaction pathways. However, the chemical inertness of methane poses significant challenges for methane activation. Compared to conventional thermocatalytic strategies, photocatalysis offer a promising alternative due to the involvement of excited states that can break the theoretical limitation of the thermodynamic equilibrium in steady states. This enables methane activation and conversion to be carried out under mild conditions. Meanwhile, transition metal oxides offer abundant lattice oxygen‐related active sites, making it easier to break methane C–H bonds during various photocatalytic methane oxidation reactions. Despite the extensive study of photocatalytic methane conversion, there is still much to explore, including advanced photocatalyst design, mechanism investigation, reaction exploration, and photoreactor optimization.

1) Photocatalyst design. Advanced photocatalyst development in the context of photocatalytic methane conversion requires consideration of several factors beyond traditional thermal catalysis. In addition to the factors such as catalytic active site, microscopic morphology, specific surface area, and surface defect, photocatalyst must also address light absorption, charge carrier migration, and various photogenerated active species.

At present, most semiconductor photocatalysts, including ZnO, TiO_2_, WO_3_, and In_2_O_3_, exhibit good photocatalytic methane conversion performance but can only absorb a small fraction of the solar spectrum, specifically ultraviolet light, which accounts for approximately 5% of sunlight. This limitation hampers the direct utilization of sunlight for photocatalytic methane conversion. Additionally, the recombination of photoexcited electrons and holes has been a persistent issue in photocatalytic reactions. Therefore, developing photocatalysts with visible light absorption capabilities and high electron‐hole separation and transfer efficiency is a crucial and ongoing research in the field of photocatalytic methane conversion.

2) Mechanism Investigation. Investigating the photocatalytic methane conversion mechanism poses significant challenges due to the ultrafast timescales involved in the process. The generation of the photogenerated electron‐hole pair occurs in the femtosecond order. While their subsequent separation and migration occur in the picosecond order, and the catalytic reaction typically takes place in the millisecond order. This multiscale transient process makes the study of reaction mechanisms extremely complex.

In photocatalytic methane conversion, there are a numerous surface adsorbed species and free radical intermediates, but their evolution and interaction remain largely unexplored. Detecting the transient active species involved in the reaction is difficult, and the nature of the reaction intermediates is poorly understood at present. In addition, understanding the interaction between the photocatalytic active sites and methane molecules (and oxidants) requires further elucidation. To advance our understanding of the mechanism behind photocatalytic methane conversion, there is an urgent need to develop appropriate in situ detection methods such as time resolved surface adsorbed species and free radical species detection. These methods will play a crucial role in further studying and revealing the intricate details of the photocatalytic methane conversion process.

3) Reaction exploration. Finding new methane conversion reactions is a crucial aspect of advancing photocatalytic methane conversion research. Currently, the main target methane conversion products include CO, CH_3_OH, HCHO, C_2_H_6_, C_2_H_4_, to name a few, with limited research on CH_3_Cl and benzene. As a promising chemical raw material, there is promising potential to use methane as an easily available methylation reagent, which could become a key development direction in the near future. In addition, utilizing methane as a starting point for constructing chemicals containing C–S, C–N, and other C–X bonds could open up new possibilities in the design and synthesis of organic molecules. Expanding the range of methane conversion reactions and products will undoubtedly contribute to the advancement of photocatalytic methane conversion and provide innovative insights for creating valuable chemical compounds with diverse applications. For example, photoelectrocatalytic methane conversion, involving the application of an external voltage to facilitate the charge transfer process, offers an alternative pathway for methane conversion. However, it is important to note that this approach is currently in its nascent stages of exploration.
4) Photoreactor design. Advanced photoreactors is a critical aspect of optimizing photocatalytic methane conversion reactions. The photoreactor should not only ensure high mass transfer efficiency of reactants but also take the light path structure and energy loss into consideration to maximize solar energy utilization. To design an efficient photoreactor, comprehensive theoretical support is required, including understanding the structural parameters of the reactor, the absorption property of photocatalysts, and the reaction kinetics of specific methane conversion reactions, among other factors.

As researchers delve further into these aspects, photocatalytic methane conversion holds great potential for practical applications in the future.

## Conflict of Interest

The authors declare no conflict of interest.
